# Effectiveness of a Motivational Nutritional Intervention through Social Networks 2.0 to Increase Adherence to the Mediterranean Diet and Improve Lung Function in Active Smokers: The DIET Study, a Randomized, Controlled and Parallel Clinical Trial in Primary Care

**DOI:** 10.3390/nu13103597

**Published:** 2021-10-14

**Authors:** Patricia Salamanca-González, Rosa Maria Valls-Zamora, Anna Pedret-Figuerola, Mar Sorlí-Aguilar, Antoni Santigosa-Ayala, Roxana-Elena Catalin, Meritxell Pallejà-Millán, Rosa Solà-Alberich, Francisco Martin-Lujan, the CENIT Research Group Investigators

**Affiliations:** 1 Functional Nutrition, Oxidation and Cardiovascular Disease Group (NFOC-SALUT), Facultat de Medicina i Ciències de La Salut, Universitat Rovira i Virgili, Sant Llorenç, 21, 43201 Reus, Spain; patricia.salamanca@urv.cat (P.S.-G.); rosamaria.valls@urv.cat (R.M.V.-Z.); anna.pedret@urv.cat (A.P.-F.); rosa.sola@urv.cat (R.S.-A.); 2 CENIT Research Group, Institut Universitari d’Investigació en Atenció Primària Jordi Gol (IDIAP JGol), Gran Via de les Corts Catalanes, 587, 08007 Barcelona, Spain; mar.sorlia@gmail.com (M.S.-A.); tsantigosa.hj23.ics@gencat.cat (A.S.-A.); recatalin.tgn.ics@gencat.cat (R.-E.C.); 3 Department of Primary Care Camp de Tarragona, Institut Català de la Salut, Doctor Mallafré Guach, 4, 43007 Tarragona, Spain; 4 Departament de Medicina i Ciències de La Salut, Universitat Rovira i Virgili, Sant Llorenç, 21, 43201 Reus, Spain; mpalleja@idiapjgol.info; 5 Research Support Unit Camp of Tarragona, Institut Universitari d’Investigació en Atenció Primària Jordi Gol (IDIAP JGol), Camí de Riudoms, 53-55, 43202 Reus, Spain; 6 Institut d’Investigació Sanitària Pere Virgili (IISPV), Hospital Universitari Sant Joan XXIII, Doctor Mallafré Guasch, 4, 43007 Tarragona, Spain

**Keywords:** Mediterranean diet, lung disease, nutritional intervention, primary care centers

## Abstract

Background: Diet can help preserve lung function in smokers, as well as aid individuals who avoid smoking. This study aimed to evaluate the effectiveness of a nutritional intervention, using the Social Networks 2.0 tool, to increase adherence to the Mediterranean diet (MD) and improve lung function in smokers without prior respiratory disease. Methods: A randomized controlled parallel design was used. The participants were assigned to either the intervention or control group. Data from representative smokers without respiratory disease (*n* = 77) aged 18–70 years were analyzed. The participants completed a validated semi-quantitative food-frequency questionnaire, and their adherence to the diet was evaluated by using the questionnaire called the Mediterranean Diet Adherence Score (MEDAS, with 14 items), which considers ≥9 points to indicate high adherence. The lung function was assessed by spirometry. Associations among variables were determined by logistic regression. Results: A comparison of the variables at the end of the study between the control and intervention groups showed that the intervention significantly increased adherence to the MD based on the MEDAS questionnaire (0.69 (2.1) vs. 2.05 (2.03); *p* = 0.009). Specifically, the consumption of fruits was increased after two years in both groups; however, a more significant increase was detected in the intervention group (121 (178) vs. 12.7 (167) in the control group; *p*-value = 0.008). In the unadjusted analysis, the intervention only showed a statistical significant increase in the score of adherence to the MD (β: 1.36; 95% CI 0.35; 2.3; *p* = 0.009), and this increase was maintained after adjusting for age and sex (β: 1.15; 95% CI 0.05; 2.2; *p* = 0.040) and after adjusting for various sociodemographic, lifestyle and anthropometric variables (β: 1.17; 95% CI 0.02; 2.31; *p* = 0.046). The pulmonary function parameters improved more in the intervention group; however, no significant differences were observed between the two groups. Conclusions: A nutritional intervention based on a dietetic-nutritional education program resulted in a significant increase in adherence to the MD. However, some evidence suggests that an MD dietary intervention can improve lung function, but in our study, we were not able to demonstrate this. Further research is needed to obtain more robust data and confirm a possible benefit of the program before it can be extended to general practice.

## 1. Introduction

Diet and nutrition have been recognized as modifiable risk factors for the development and progression of multiple chronic diseases, including lung diseases [1,2]. Although the fundamental message in public health regarding lung diseases continues to be smoking cessation, the multifactorial nature of many chronic lung diseases opens up the possibility of intervening in other modifiable risk factors, such as nutrition [3].

The MD is a dietary pattern characterized by the consumption of large quantities of vegetables, legumes, fruits, cereals, preferably whole grains and extra virgin olive oil, which ensures a good supply of fiber, antioxidants, phytosterols, polyphenols and unsaturated fatty acids [4]. These food categories are rich in antioxidants, such as vitamin C, which is present in many fruits and vegetables, and vitamin E, which is present in olives. High adherence to MD has been linked with higher plasma concertation of these antioxidants [5]. Vitamin C is one of the most studied nutrients for its relation with lung health and has been linked with higher lung function among chronic obstructive pulmonary disease (COPD) patients and healthy adults [6]. Data from some studies have shown a positive relationship between vitamin E intake and forced vital capacity (FVC) and forced expiratory volume in 1 s (FEV1) [7].

The MD is also accompanied by high intake of omega-3 fatty acids, through weekly fish consumption, which provides anti-inflammatory action. The antioxidant and anti-inflammatory properties of MD have been linked with many health benefits; for instance, they offer protection against metabolic diseases, such as diabetes mellitus, cardiovascular disorder and hyperlipidemia, as well as against many types of cancer or even neurodegenerative diseases. Since MD protects against cell oxidation and inflammation in several systems, it is reasonable to consider that these effects would also apply for the lung tissues [8].

In particular, the intake of fruits and vegetables is highly associated with respiratory health due to the benefits of antioxidant vitamins (C, D, E and ß-carotene) [9,10], minerals (magnesium, calcium, selenium and potassium) [11] and several phytochemical compounds and dietary fibers present in these plant-based foods [12,13]. Moreover, some evidence shows that the consumption of omega-3 fatty acids, mainly eicosapentaenoic acid (C20: 5) and docosahexaenoic acid (C22: 6), which are mainly found in fatty fish and shellfish interfere with the body’s inflammatory response and may prevent some of the inflammatory mechanisms involved in the pathophysiology of various lung diseases [14,15,16]. Conversely, the high consumption of red and processed meat is associated with poorer lung function and an increased risk of COPD [17,18,19]. In addition, excessive alcohol consumption has also been shown to exert detrimental effects on lung function, although the specific threshold remains undefined [20,21,22,23,24].

In a previously published study by our team [25], we identified three dietary patterns associated with lung function: a Mediterranean-type pattern, a Westernized pattern and an alcohol consumption pattern. In the adjusted multivariate model, the Mediterranean-type pattern was associated with healthier lung function compared to the other two dietary patterns identified, especially in women (OR 0.71 [95% CI 0.28–1.79], OR 5.62 [95% CI 1.17–27.02] and OR 11.4 [95% CI 2.25–58.47], respectively). Our group also previously reported on the feasibility of conducting a randomized controlled clinical trial to assess the efficacy of a dietary intervention supported by the Social Networks 2.0 tool in primary health care settings [26].

This program was conceived as a comprehensive form of medical and healthcare focused on the patient, and the involved individuals (patients, professionals, administrators and providers) participate actively by using Social Networks 2.0 and Web 2.0 tools to improve the quality of life of communities. Currently, social networks are a form of interaction and have had a strong impact on new forms of communication. These networks are supported by so-called social media and based on the functions and properties of Web 2.0 tools; the network processes are based on the internet [27].

Following recommendations for good practice when designing feasibility and pilot studies, we aimed to evaluate the effectiveness of a nutritional intervention incorporating the Social Networks 2.0 tool to increase adherence to the MD and assess its effects on lung function in smokers without prior respiratory disease.

## 2. Materials and Methods

According to CONSORT guidelines, our study protocol was registered and provided a detailed overview of the methodology before initiation of the study (ClinicalTrials.gov; Identifier, NCT02151669; 26 May 2014).

### 2.1. Study Design

A randomized, controlled, parallel design was used. The participants were assigned to either the intervention group or the control group by an external investigator. However, given the open nature of the intervention, blinded assignment of the participants and researchers was not feasible.

The DIET study follows the principles set out in the Declaration of Helsinki and the ethical and scientific quality standard of the International Conference on Harmonization guidance E6 (R2) for Good Clinical Practice. It also fulfils the requirements established in the legislative framework in Spain for the field of biomedical research, the protection of personal data and bioethics. The ethical and scientific quality of the study was previously evaluated by the Ethical and Scientific Committees of the Research Support Unit of the Primary Care Research Institute-IDIAP Jordi Gol (reference number 4R13/068, approved 10 December 2013).

### 2.2. Selection of Participants and Randomization

The study population was recruited from patients who visited primary health-care centres of Catalan Institute of Health (ICS, for its Spanish acronym) in the Tarragona district of Catalonia (Spain). The inclusion criteria were men and women aged 35 to 70 years (both inclusive), current smokers with cumulative consumption ≥10 pack-years, internet access and ability to use new technologies (or having someone available to provide relevant assistance). The exclusion criteria were a previous diagnosis of any respiratory disease (such as COPD, asthma, bronchiectasis, pulmonary fibrosis, etc.), any chronic or terminal condition that could affect the baseline parameters (such as cancer, severe heart or cerebrovascular disease, liver disease or kidney failure), or any reason that, at the discretion of the researcher, might make it difficult to follow-up the participant during the study period (such as mental illness).

During recruitment, all individuals received information about the study objectives and the activities related to their participation and signed an informed consent form prior to their inclusion.

### 2.3. Procedures

The participants were assigned randomly to the control group or the intervention group (1:1), using a centralized process carried out by the Research Support Unit of the IDIAP Jordi Gol based on a simple random number list generated with a computer for this purpose with the EPIDAT 3.0 program (Dirección Xeral de Saúde Pública da Consellería de Sanidade; Xunta de Galicia; Spain).

All the participants attended an initial visit consisted of various medical questionnaires of interest and a unified data collection notebook. The complete information contained in the Data Collection Notebook is shown in Supplementary Materials Table S1. In summary form, the Data Collection Notebook includes sociodemographic data, pathological history, smoking habit and clinical examination, which consisted of a basic analytical electrocardiogram, measurement of the expired carbon monoxide levels (expired-CO), and a forced spirometry with bronchodilator test according to the recommendations of the American Thoracic Society and The European Respiratory Society [28]. The dietary-nutritional history of the patient was also obtained, and this history included the results from a questionnaire related to diet and lifestyle, a questionnaire consisting of 14 items related to adherence to the Mediterranean diet (MEDAS) [29] and a food-frequency questionnaire consumption (weekly or monthly during the last year) [30]. The MEDAS questionnaire assigns one point to each item and classifies adherence to the MD as low, moderate or high based on the final score. In addition, a survey of physical activity in leisure time collected the physical activities performed by the participants in the last month and in the last year and the time spent in each activity. The validated version for the Spanish population of the Minnesota Leisure Time Physical Activity Questionnaire [31] and the physical activity classification, which was measured with the short Catalan version of the International Physical Activity Questionnaire (IPAQ) [32], were used.

All variables were determined at baseline and at the two-year study follow-up visit. All the information was collected in a computerized database, which was accessible only to the study investigators. Access was restricted and controlled by a personal password for each researcher, who was responsible for entering the data of the participants.

### 2.4. Intervention Group

During the initial visit, the intervention group started the “Programme of Dietetic-Nutritional Education in Mediterranean Diet in Primary Care Centres” applied by a nutritionist and kept a blog (Blog 2.0) for 2 years. This motivational nutrition program was based on an individual intervention to explain the MD pattern and the benefits of following the program.

The MD pattern was described based on the recommendations generated by the Mediterranean Diet Foundation’s Pyramid, the guide for adult populations [33]. This graphical representation was conceived as a simplified pyramid, to be adapted to the specific realities of different countries (e.g., portion sizes) and variations in dietary pattern related to the diverse geographical, socioeconomic and cultural contexts of the Mediterranean region. During the intervention visit, the MD pyramid was used as educational material to discuss the results of the dietary questionnaires. Various educational materials showing food groups adapted to the habits and personal preferences of each participant were also used. The main aim of the educational material was to modify the dietary pattern as a whole, rather than focusing on changes in specific foods or micronutrients.

During this visit, the intervention group was also given access to the Blog 2.0 with a personal username and password. This blog was designed ad hoc by the team and its use was exclusive to the study. Participants were advised to log in weekly to get updated information. On the blog they could find information about the different foods that characterize MD and their benefits, cooking habits, modified recipes, examples of standard healthy menus and other topics of interest related to healthy habits. This Blog 2.0 information was updated regularly and its current content can be found at the following address: https://estudimedistar.wordpress.com (accessed on 30 September 2021).

### 2.5. Control Group

During the initial visit, the participants in the control group received written dietary recommendations appropriate to their clinical–metabolic situation (advice to control weight, glycaemia, cholesterol, blood pressure, etc.) but did not have access to Blog 2.0 or personalized recommendations regarding the MD.

### 2.6. Study Variables

The main variables of interest are the adherence to the MD and lung function. Adherence to the MD was defined based on the MEDAS questionnaire, and ≥9 points was considered to reflect high adherence [34]. Lung function was defined according to the guidelines of the American Thoracic Society and The European Respiratory Society [35]: An anomalous FVC or FEV1 was defined as a value lower than 80% of the predicted value. In addition, an FEV1/FVC ratio <70% was considered to reflect an alteration. Significant changes in the FVC and FEV1 values between the initial and final visits (>12% and 200 mL) were also considered as decreases beyond what is expected in smokers.

### 2.7. Statistical Analyses

Our sample size was pragmatic, with the aim of including sufficient participants to ensure an alpha risk of 0.05 and a beta risk of 0.2 in a two-sided test, recognizing ≥2 units in the MEDAS [34] as indicating a statistically significant difference, and assuming that the common standard deviation was 3 units. Therefore, based on an anticipated dropout rate of 10%, 40 subjects were allocated to the intervention group, and 40 other participants were assigned to the control group. This sample size, 40 participants in each group, was sufficient to observe significant differences in lung function between the groups (decrease in FEV1 > 10–12 mL/year) [36,37]. The calculations were based on an online tool: Sample size and power calculator GRANMO (Available at: https://www.imim.es/ofertadeserveis/software-public/granmo/. Accessed on 30 September 2021).

Categorical variables are described as frequencies or percentages, and quantitative variables are described as means and standard deviations or medians and 1st quartiles and 3rd quartiles, depending on whether they are distributed normal or not, respectively. A comparison between the intervention group and control group of the baseline values and an analysis of the changes over 2 years were made by using the χ^2^ test for categorical variables and the Student’s *t*-test or Mann–Whitney U test for quantitative variables, depending if they were normally distributed or not, respectively. For paired comparisons of qualitative variables, McNemar’s test was used.

The difference was calculated by comparing the data obtained from the questionnaires on adherence to the MD and FFQ obtained at the first visit with those obtained at the final visit of the study. In addition, to determine the relationship between the groups for each outcome (variables of lung function and points of adherence to the MD), a multivariate analysis was performed by using linear regression. We obtained three models for each outcome: unadjusted, adjusted by age and sex and adjusted by sociodemographic data, smoking habit, physical activity and anthropometric data.

All analyses were performed by using the statistical package R (R Foundation for Statistical Computing, Vienna, Austria; version 4.0.5), according to the intention-to-treat analysis principle and considering a significance level with a *p*-value < 0.05.

## 3. Results

**Figure 1:**
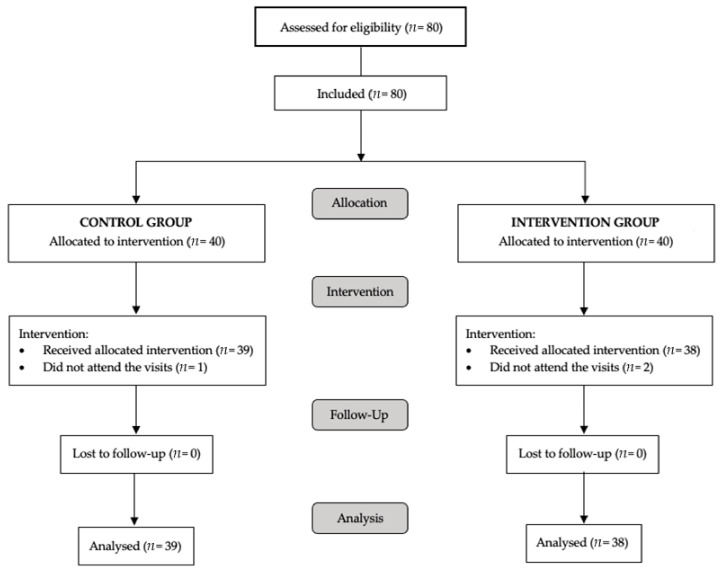
Flow diagram of the project: participant selection, randomization and follow-up.

A total of 77 participants were included and received intervention; the participants had an average age of 53.5 (9.6) years, and 71.4% of the participants were women. The cumulative tobacco consumption of all the participants was 23 pack-years, with a daily consumption of 13.5 [10.0; 20.0] cigarettes and a low degree of physical activity (5.7 [2.71; 11.3] hours/week). No significant differences were found between the groups except in age (57.0 (8.81) years in the control group vs. 50.0 (9.34) years in the intervention group; *p* = 0.001). All sociodemographic data of the participants are shown in Supplementary Materials Table S2. A flow diagram of the study is shown in Figure 1.

Table 1 shows the baseline values and the change after two years obtained for the study population divided by groups. In relation to the anthropometric variables and the results of the laboratory tests and spirometry, it should be noted that the metabolic profile values were optimal and that those related to lung function were within normal values. At baseline, we observed only significant differences in the diastolic blood pressure (80.0 (7.5) in the control group and 75.4 (8.6) in the intervention group; *p* = 0.018). At the end of the study, we observed significant differences in the total cholesterol (+0.65 mg/dL [−35.13; 15.5] in the control group and −8.85 mg/dL [−15.08; 14.7] in the intervention group; *p* = 0.046) and LDL cholesterol (−5.51 mg/dL [−40.92; 8.12] in the control group and −10.37 [−20.07; 3.92] in the intervention group; *p* = 0.012) levels.

In addition, significant differences were not observed regarding adherence to the MD at baseline, which can be considered moderate, and no differences were found between the two groups (8.6 (2.9) points for the control group and 7.9 (1.6) points for the intervention group; *p* = 0.092). The analysis of the variables at the end of the study showed a significant increase in adherence to the MD based on the MEDAS questionnaire between the control group and the intervention group (0.69 (2.1) vs. 2.05 (2.03); *p* = 0.009). We also observed an improvement in values measured by using laboratory tests, physical examinations and variables related to lung function, but none of the improvements were statistically significant.

**Figure 2:**
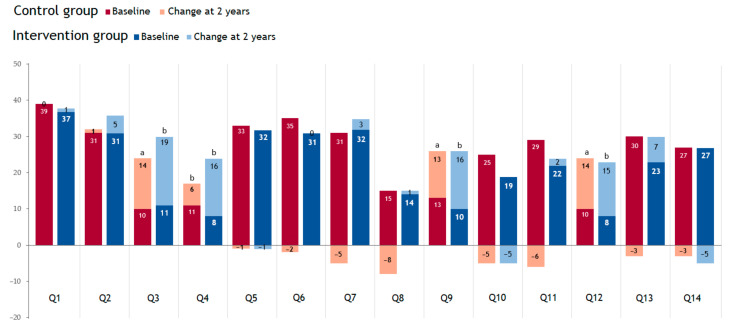
Participants with positive answer (%) to each of the 14 Items of MEDAS. MEDAS refers to the 14-point test of adherence to the Mediterranean diet validated by the PREDIMED study [34]: (Q1) use olive oil as main culinary fat, (Q2) olive oil > 4 tablespoons, (Q3) vegetables ≥ 2 servings/day, (Q4) fruits ≥ 3 servings/day, (Q5) red or processed meats < 1/day, (Q6) butter/cream/margarine < 1/day, (Q7) soda drinks < 1/day, (Q8) Wine glasses ≥ 7/week, (Q9) legumes ≥ 3/week, (Q10) fish or seafood ≥ 3/week, (Q11) commercial bakery ≤ 2/week, (Q12) nuts ≥ 3/week, (Q13) poultry more than red meats and (Q14) use of sofrito sauce ≥ 2/week. Data are presented with the number of answer participants’ (*n*). Negative values show the difference in the number of participants responding affirmatively to the questionnaire question at the final visit compared to the baseline visit. The McNemar test was used for analysis (test for paired qualitative variable). ^a^ *p*-value <0.05 when comparing data between baseline and final visit. ^b^ *p*-value <0.01 when comparing data between baseline and final visit.

Figure 2 shows the positive answers given by the participants in the 14-item MEDAS questionnaire. The information shows how the diet of both groups improved significantly after two years of intervention, as indicated by the questions related to the consumption of vegetables and fresh fruits and the consumption of legumes and nuts. Furthermore, the control group exhibited a significant decrease in red wine consumption. The consumption of the other foods did not show significant differences at the end of the study in any of the study groups.

Table 2 shows the result from the comparison of food consumption between the groups. At the beginning of the study, significant differences in fruit consumption (300 [150; 300] in the control group vs. 136 [66.1; 236] in the intervention group; *p*-value = 0.021) were found. Moreover, the consumption of fruit increased after two years in both groups, but a marked increase was found in the intervention group (121 (178) in the intervention group vs. 12.7 (167) in the control group; *p*-value = 0.008). Although the results showed increases in the consumption of vegetables, dairy products, eggs, legumes, nuts and shellfish and a slight decrease in the consumption of cereals and meat products, the consumption of the rest of the foods did not show significant differences at the end of the study in any of the study groups.

Finally, to analyze the efficacy of the intervention on the changes in diet and lung function, a linear regression was performed, as shown in Table 3. In the unadjusted analysis, the intervention only showed statistical significance in relation to the score reflecting adherence to the MD (β: 1.36; 95% CI 0.35; 2.3; *p* = 0.009), and this significant association was maintained after adjusting for the variables age and sex (β: 1.15; 95% CI 0.05; 2.2; *p* = 0.040) and after adjusting for various sociodemographic, lifestyle and anthropometric variables (β: 1.17; 95% CI 0.02; 2.31; *p* = 0.046). Regardless of this finding, the intervention did not induce significant changes in lung function.

## 4. Discussion

This novel study investigated the feasibility of a nutritional intervention to increase adherence to the MD and its impact on lung function in smoking patients in primary care centers in the Tarragona health area in Catalonia, Spain. Their results demonstrate that a nutritional intervention based on a dietetic–nutritional education program, and enhanced through Social Networks 2.0, induces a significant increase in adherence to the MD, although a significant impact on lung function was not observed.

Previous studies have provided evidence showing that higher adherence to the MD improves parameters related to lung function [4]. A recent review reported that a healthy diet, such as DM, could be helpful in improving lung function in patients with respiratory diseases [38]. However, the existence of an association in smoking patients without previous respiratory disease has not yet been demonstrated. Currently, specific interventions to promote an adequate dietary lifestyle in relation to lung health are not fully established. Other studies confirmed the effectiveness of the use of various similar interventions related to the use of social networks in several chronic diseases, such as diabetes, obesity or metabolic syndrome [39,40,41,42,43,44,45,46,47,48,49,50]. This study constitutes the first clinical trial that used Blog 2.0 and involves a smoking population without previous respiratory disease in primary care centers. Previous studies have shown that both a face-to-face dietary intervention and a simple, non-intensive, web-based intervention are useful to increase adherence to DM in the general population [40,41,42] and, therefore, to improve different chronic diseases [43]. For example, several studies aimed to decrease obesity in the population through a web-based intervention with email and/or text message alerts [44,45,46]. On the other hand, there are some studies related to diabetes mellitus [47,48], where the intervention was delivered through an internet-based educational program, together with explanatory videos and group sessions. In addition, interventions aimed at improving diseases related to the digestive tract were found [49,50] wherein internet-based educational interventions and group discussions were used. The blog contents of the present study are part of an educational intervention on DM, which, compared to those described above, is less intensive. However, this type of intervention, being simple and non-intensive, makes it more feasible in most settings and easier to extend to the general population.

Our previous study, a Westernized dietary pattern and a diet with high alcohol consumption was found to be associated with impaired spirometry values and a higher prevalence of abnormal lung function compared with the results found for participants with adherence to a dietary Mediterranean-like pattern [25]. In addition, the possible beneficial effects of the MD on lung function have been revealed in cross-sectional studies performed with smokers and subjects with different related diseases, such as asthma and COPD [51,52,53]. However, caution should be exercised because no intervention study has been reported thus far, and to our knowledge, no direct evidence on their effects on lung function in current smokers has been published. In any case, understanding the impact of the diet on maintaining lung function can increase awareness of the importance of nutritional effects beyond avoiding smoking, as well as provide instructions for future research and strategies to promote lung health and prevent the onset and progression of lung diseases [2,25].

Regarding the effect of diet on lung, although analyzing the effect of individual foods has been valuable, there remain conceptual and methodological limitations because actual meals consist of nutrients that are likely to interact or exhibit synergy with each other [54]. Therefore, dietary patterns provide a framework for examining the health effects of an entire diet. [55,56]. In parallel, dietary quality indices or indicators (DQI) are algorithms that aim to evaluate the overall diet and categorize individuals according to the extent to which their eating behavior is healthy or unhealthy. Predefined indices assess dietary patterns based on current nutrition knowledge and they have been developed primarily for nutritional epidemiology to assess dietary risk factors for noncommunicable diseases [57]. Similarly, the Healthy Eating Index (HEI), the Diet Quality Index (DQI), the Healthy Diet Indicator (HDI) and the Mediterranean Diet Adherence Score (MEDAS) are the four original diet quality scores that have been developed and most extensively validated [58,59]. Evaluating the dietary habits through questionnaires such as MEDAS, which has 14 items, could provide information about how our dietary habits influence lung health.

As we have commented previously, a dietary pattern characterized by a high intake of refined grains, processed and red meats, desserts, sweets and fried foods is positively associated with an increased risk of pulmonary function diseases, such as COPD or asthma, even after adjusting for age and total energy intake [60,61]. On the other hand, a diet rich in vegetables, fruits, fish, and whole grains is associated with better lung function in smoking men [62,63]. Recently, a loss of adherence to healthy diets, such as the MD, causing a decrease in the consumption of fruits, vegetables, whole grains and fish and a greater consumption of processed and refined foods, has been suggested to contribute to the higher prevalence of chronic diseases, including lung disease, mainly in developed countries [64,65].

Our results show that a personalized, non-intensive and simple nature of a dietary-nutritional intervention, enhanced through Social Networks 2.0, helps improve adherence to the MD. In the present study, we observed that the dietary pattern of the group that showed higher adherence to the MD was characterized by a higher consumption of legumes, fish, shellfish, eggs, vegetables, fruits, nuts, dairy products, cereals and white meat. At the end of the study, although only fruit consumption showed a significant difference, an increase in the consumption of the abovementioned foods was observed. In contrast, lower consumption of red and processed meat, alcoholic beverages, sugary drinks, light drinks and processed foods was observed in general. In turn, we found that the levels of the variables related to lung function tended to improve. Previous studies have also provided evidence of protective action on pulmonary ventilation parameters, such as FVC and FEV1 [66,67]. This finding can be explained by the presence of natural antioxidants and fatty acids in addition to a high content of dietary fiber in foods, such as nuts, which appears able to neutralize the harmful effect of tobacco through the action of various enzymatic antioxidants, including superoxide dismutase, catalase and glutathione peroxidase [68,69,70].

The present study has several strengths. Our clinical trial constitutes the first analysis of the association of dietary patterns with lung function in the smoking population without previous respiratory disease in primary care settings and thus provides new information that could lead to the clinical application of dietary modification, using the Social Networks 2.0 as a tool to preserve lung function and promote smoking cessation. Furthermore, in our study, we have a low loss to follow-up, which reinforces our results. The explanation for this high follow-up rate could be that the data were collected in primary care centers. In Spain, attendance at primary care centers is high (>5 times per year), which could facilitate the follow-up of the study patients [71]. In addition, all patients were followed by the nutritionist, who was in charge of the follow-up at all times during the study. Finally, compared to previous studies, this study provides objective measures for the assessment of lung function through spirometry and post-bronchodilator tests that avoid possible bias.

We must also consider some limitations. Consistent with recommendations on the analysis of feasibility studies, the conclusions should be interpreted with caution because the results may be modified by conducting large-scale studies. As a main limitation, we must mention the sample size of this study and the limited study duration. Our sample size was pragmatic, with the aim of including sufficient patients to confirm the variability of the proposed intervention and a possible impact on lung function. The intervention was generally well received, but time was the main barrier to its use. The short intervention time may also have limited the statistical power to detect some differences between the consumption patterns of different foods and the values of the variables related to lung function. Therefore, as previously suggested [25], there is a need to incentivize participation and compensate participants for the time needed to participate in a definitive trial of the intervention. Particularly if the intervention is to be translated into routine practice, the question of which professional will apply the program should be considered. Furthermore, to demonstrate these beneficial effects, a longer intervention time in a larger study population are needed. Currently, our CENIT research group is working on a clinical trial called MEDISTAR [72] with the aim of confirming these results. Their results may open up the possibility of establishing a nutritional intervention, together with the fundamental recommendation to stop smoking, in this group of at-risk populations.

## 5. Conclusions

In conclusion, our findings show that the nutritional intervention tested in our primary care centre is feasible and effective for increasing adherence to the MD and improving the dietary pattern of the population. Although some evidence suggests that an MD dietary intervention can improve lung function, in our study, we were not able to demonstrate this. Therefore, further appropriate studies are needed to obtain more robust data to confirm the potential benefit of this intervention to improve the diet of smokers before extending the recommendation to general practice.

## Figures and Tables

**Table 1:** Baseline and change at two-year follow-up characteristics of the study population, compared by groups.

	Baseline		Change at 2 Years	
	Control Group	Intervention Group	Control Group	Intervention Group
	(*n* = 39)	(*n* = 38)	(*n* = 39)	(*n* = 38)
**Health habits**				
Physical activity, hours/week	6.50 [3.12;11.6]	5.25 [2.36;11.0]	0.82 (6.35)	2.09 (5.48)
Current smoking (%)	100	100	2 (5.13)	5 (13.2)
Current consumption, cigarettes/day	15.0 (10.0; 20.0)	12.0 (10.0; 20.0)	0.00 [0.00; 0.00]	0.00 [0.00; 0.00]
Mediterranean Diet Adherence Score (MEDAS) ^b^	8.6 (2,9)	7.9 (1,6)	0.69 (2.14)	2.05 (2.03)
**Anthropometrics variables**				
Height, cm ^a^	158 (7.7)	163 (10.1)	-	-
Weight, kg	68.2 (12.2)	71.4 (15.1)	−1.69(6.18)	1.98(4.89)
BMI, kg/m	27.2 (4.5)	26.9 (5.01)	−0.80 (2.22)	−0.93 (1.78)
Waist circumference, cm	87.2 (10.5)	88.3 (12.0)	−0.79 (8.46)	−3.66 (6.64)
**Clinical variables**				
SBP, mmHg	129 (26.7)	128 (14.5)	0.18 (24.2)	−0.03 (16.4)
DBP, mmHg ^a^	80.0 (7.5)	75.4 (8.6)	−0.39 (6.83)	3.08 (11.5)
**Biochemical variables**				
Total Cholesterol, mg/dL ^b^	209 (49.20)	197 (35.10)	0.65 [−35.13; 15.5]	−8.85 [−15.08; 14.7]
HDL—Cholesterol mg/dL	61.1 (19.4)	55.2 (15.0)	1.05 [−5.47; 8.98]	2.80 [−4.20; 7.00]
LDL—Cholesterol, mg/dL ^b^	131 (36.6)	116 (34.1)	−5.51 [−40.92; 8.12]	−10.37 [−20.07; 3.92]
Triglycerides, mg/dL	129 (64.3)	132 (91.9)	3.00 [−7.00; 28.0]	−9.50 [−37.50; 51.0]
Glucose, mg/dL	90.8 (23.0)	98.8 (33.1)	2.00 [−1.00; 13.0]	−1.00 [−8.00; 12.0]
**Pulmonary function variables**				
FVC, mL	3340 (850)	3590 (1000)	−120 [−180; −50]	−70 [−320; 10]
FVC, (%)	97.7 (16.2)	93.3 (16.5)	0.00 [−2.50; 0.00]	0.00 [−6.00; 1.00]
FEV1, mL/min	2630 (600)	2820 (890)	−140 [−210; −50]	−90 [−210; 10]
FEV1, (%)	103 (16.4)	95.7 (19.0)	0.00 [−7.00; 0.00]	0.00 [−4.00; 1.00]
FEV1/FVC ratio, (%)	79.3 (6.26)	78. (6.42)	−0.68 [−1.15; −0.25]	−0.55 [−0.84; −0.16]

Data are presented with frequencies and (%), the mean (standard deviation) or as median [Q1; Q3], according to the type of variable. The Shapiro–Wilk test was used to decide the normality of the variables with a significance of 0.01. MEDAS, 14-point test of adherence to the Mediterranean diet validated by the PREDIMED study [34]; BMI, body mass index; SBP, systolic blood pressure; DBP, diastolic blood pressure; HDL, high-density lipoprotein; LDL, low-density lipoprotein; FVC, forced vital capacity; FEV1, forced expiratory volume in 1 s. ^a^ *p*-value < 0.05 when comparing data between groups in baseline visits ((baseline visit of control group) vs. (baseline visit of intervention group)). ^b^ *p*-value < 0.05 when comparing data changes between groups during study ((final visit—baseline visit of the control group) vs. (final visit—baseline visit of the intervention group)).

**Table 2:** Baseline and change at two years—consumption of different food (g/day or mL/day) of the study population, compared by groups.

	Baseline		Change at 2 Years	
	Control Group	Intervention Group	Control Group	Intervention Group
	(*n* = 39)	(*n* = 38)	(*n* = 39)	(*n* = 38)
**Cereals, nuts and legumes**				
Legumes (g/day)	10.0 [10.0; 20.0]	10.0 [10.0; 20.0]	10.0 [0.00; 20.0]	10.0 [0.00; 20.0]
Nuts (g/day)	4.29 [0.00; 10.7]	4.29 [0.00; 12.9]	0.00 [0.00; 12.9]	12.9 [0.00; 19.6]
Cereals (g/day)	163 (62.6)	181 (63.4)	−0.72 (63.0)	−13.29 (70.1)
**Fruits and vegetables**				
Vegetable (g/day)	194 (119)	152 (68.2)	21.4 [0.00; 72.9]	55.0 [14.3; 103]
Fruits (g/day) ^a,b^	300 [150; 300]	136 [66.1; 236]	12.7 (167)	121 (178)
Milk and dairy products				
Dairy products (g/day)	261 [225; 475]	282 [226; 475]	35.7 [0.00; 135]	36.8 [0.00; 177]
**Meat, fish and eggs**				
White meat (g/day)	32.9 [16.4; 49.3]	32.9 [16.4; 49.3]	0.00 [0.00; 8.21]	0.00 [−16.43; 0.00]
Red meat (g/day)	20.7 [17.5; 35.0]	31.4 [20.7; 43.0]	0.00 [−6.43; 2.14]	0.00 [−13.75; 5.89]
Processed meat (g/day)	16.4 [6.34; 20.7]	18.6 [15.0; 22.3]	4.29 [−5.00; 18.6]	1.07 [−7.50; 9.55]
White fish (g/day)	38.6 [19.3; 38.6]	38.6 [38.6; 38.6]	0.00 [0.00; 0.00]	0.00 [0.00; 0.00]
Blue fish (g/day)	38.6 [19.3; 38.6]	38.6 [19.3; 38.6]	0.00 [0.00; 0.00]	0.00 [0.00; 0.00]
Shellfish (g/day)	4.82 [0.00; 19.3]	19.3 [1.21; 19.3]	0.00 [0.00; 19.3]	0.00 [0.00; 19.3]
Eggs (g/day)	25.7 [12.9; 38.6]	25.7 [12.9; 25.7]	12.9 [0.00; 12.9]	12.9 [0.00; 12.9]
**Processed products**	15.7 [5.71; 21.4]	15.7 [10.0; 21.1]	1.43 [−6.96; 15.2]	0.00 [−9.64; 9.29]
**Pastries products**	24.3 [14.6; 41.2]	30.0 [12.9; 83.9]	−4.29 [−14.82; 4.55]	−4.64 [−41.92; 7.14]
Carbonated drinks				
Sugary and light drinks (mL/day)	0.00 [0.00; 157]	28.6 [0.00; 78.6]	0.00 [0.00; 0.00]	0.00 [−28.57; 0.00]
**Alcohol drinks**				
**Wine (mL/day)**	28.6 [0.00; 100]	14.3 [0.00; 71.4]	0.00 [0.00; 14.3]	0.00 [−14.29; 0.00]
**Fermented drinks (mL/day)**	28.6 [0.00; 100]	14.3 [0.00; 67.9]	0.00 [0.00; 14.3]	0.00 [−14.29; 0.00]

Data are presented with the grams per day of the diet by study inclusion groups, the mean (standard deviation) or as median [Q1; Q3], according to the type of variable. The Shapiro–Wilk test was used to decide the normality of the variables with a significance of 0.01. ^a^ *p*-value < 0.05 when comparing data between groups in baseline visits [(baseline visit of control group) vs. (baseline visit of intervention group)]. ^b^ *p*-value < 0.05 when comparing data changes at two years between groups ((final visit—baseline visit of the control group) vs. (final visit—baseline visit of the intervention group)).

**Table 3:** Relationship between adherence to Mediterranean diet and pulmonary function variables to randomized assignment group (unadjusted, age/sex-adjusted and multivariable-adjusted β-coefficients and 95% CI).

	Unadjusted		Age/Sex-Adjusted		Multivariable-Adjusted	
	β (95% CI)	*p*-Value	β (95% CI)	*p*-Value	β (95% CI)	*p*-Value
Mediterranean Diet Adherence ^1^	1.36 (0.35; 2.37)	0.009	1.15 (0.05; 2.25)	0.040	1.17 (0.02; 2.31)	0.046 *
Pulmonary Function						
FVC	2.18 (−2.46; 6.84)	0.352	1.65 (−3.40; 6.71)	0.517	1.22 (−4.17; 6.61)	0.652
FEV1	2.70 (−1.56; 6.95)	0.211	2.24 (−2.36; 6.85)	0.335	1.93 (−3.00; 6.86)	0.437
Ratio FVC/FEV1	−0,09 (−2.66; 2.48)	0.944	0.08 (−2,70; 2.87)	0.953	0.52 (−2.48; 3.52)	0.730

β, regression coefficient for each exposure variable; CI, confidence interval; FVC, forced vital capacity; FEV1, Forced Expiratory Volume in 1 s; ^1^ 14-point test of adherence to the Mediterranean diet validated by the PREDIMED study [34]; * *p*-value < 0.05 in a model adjusted for sociodemographic, lifestyle and anthropometric variables (age, sex, marital status, number of children, level education, level employment, body mass index, cumulative smoking and abstinence at the end of the study).

## Data Availability

All data are considered confidential and treated according to Regulation 2016/679 of the European Parliament and Council of 27 April 2016 on Data Protection and Organic Law 3/2018, of 5 December, on the protection of personal data and guarantee of digital rights. Access is restricted to the research team by password. Data are available on reasonable request. The full dataset and statistical code are available from the corresponding (fmartin.tgn.ics@gencat.cat).

## Supplementary Materials

The following are available online at https://www.mdpi.com/article/10.3390/nu13103597/s1. Table S1: Information collected through Data Collection Notebook in all visits of the study. Table S2: Sociodemographic data of the study participants.
