# Supplementary material for: Effectiveness of a Motivational Nutritional Intervention through Social Networks 2.0 to Increase Adherence to the Mediterranean Diet and Improve Lung Function in Active Smokers: The DIET Study, a Randomized, Controlled and Parallel Clinical Trial in Primary Care
Source: Nutrients. 2021 Oct 14;13(10):3597. doi: 10.3390/nu13103597 (PMC8538243; doi:10.3390/nu13103597)
Supplement: Supplementary file 1 [file nutrients-13-03597-s001.zip › nutrients-1392560-supplementary.pdf]

Supplementary Materials

Table S1. The information collected through Data Collection Notebook in all visits of the study.

**Data Collection Notebook**
Affiliations and sociodemographic data
Sex and age, civil status, number of children, education level and employment status.
Medical history with treatment received
Respiratory symptomatology (such as dyspnea, cough, expectoration, chest pain and other symptoms), clinical morbidities and treatment received.
Smoking habits
Cumulated consumption (packs-year, calculated as years of smoking multiplied by the average daily consumption of 20-cigarette packs), current consumption (cigarettes/day), nicotine dependence (Fagerström test score), motivation to quit smoking (Richmond test score), and stage of change according to Prochaska and DiClemente model (precontemplation, contemplation, preparation, action, maintenance and relapse).
Health habits
Consumption of alcohol measured with frequency and grams of alcohol/week.<br>Physical activity classification, measured with the short Catalan version of the International Physical Activity Questionnaire (IPAQ). According to the IPAQ guidelines, the participants were classified as engaged in vigorous physical activity, moderate physical activity and low physical activity.
Food consumption frequency and Mediterranean diet adherence
Food consumption frequency and dietary intake information, collected using a 45-item food frequency questionnaire (FFQ), validated for the Spanish population. The questions include the average frequency of food consumption using specific categories for number of times/week and times/month, during the previous 12 months.<br><br>Mediterranean diet adherence measured by administration of a previously validated 14-item questionnaire (MEDAS). The questionnaire consists of 12 food consumption frequency questions and two about food intake habits considered characteristic of the MD. Each question scores 0 or 1, consequently, total score ranges from 0 to 14. The higher the score, the greater the degree of adherence to MD pattern. Therefore, a score of = or >9 is defined as "high" MD adherence.
Anthropometric values and adiposity markers
Height and body weight, measured when the participants wore indoor clothing without shoes. The waist circumference, measured at the mid-point between the lower border of the rib cage and the iliac crest, according to WHO-2011 recommendations. The body mass index (BMI), calculated according to the following formula: weight (kg)/height (m^2). The waist circumference/height ratio. The conicity index, calculated according to the following equation: waist circumference/(0.109 × square root of (weight/height).
Physical examination
The blood pressure, measured twice in sitting position on the right arm, calculated as the mean value of the two measurements (using Omron M6; Omron Healthcare Europe, Hoofddorp, The Netherlands).

The 12-lead electrocardiographic record (using PageWriter TC20; Philips Medical Systems, Andover, USA).<br>The expired carbon monoxide concentration, determined by CO-oximetry (using MicroCO™; Medical Device Depot, Ellicott City, USA), that detects levels range of 0–100 ppm, with a sensitivity of 1 ppm.
Pulmonary function
The forced vital capacity (FVC), the forced expiratory volum in 1 second (FEV1), and the relationship between them (FEV1/FVC ratio), measured by spirometry (using DATOSPIR-600©; SIBELMED, Barcelona, Spain) with a disposable Lilly-type transducer). The standard procedure is performed in agreement with the ATS/ERS recommendations.
Bioquimical variables
Levels of glucose, total cholesterol and fractions, triglycerides, creatinine and transaminases were measured by standardized methods on an autoanalyzer (Beckman Coulter-Synchron, Galway, Ireland) in serum samples.

Table S2. Socio-demographic data of the study participants.

	**Baseline**	
	**Control group**	**Intervention group**
	**(n=39)**	**(n=38)**
**Sex**		
Women, n (%)	30 (76,9%)	25 (65,8%)
Age (years) , n (%)	57.0 ± 8,8	50.0 ± 9,3
**Marital status**		
Single, n (%)	26 (66,7%)	23 (60,5%)
No singles, n (%)	13 (33,3%)	15 (39,5%)
**Children**		
No children, n (%)	9 (23.1%)	14 (36.8%)
≥ 1children, n (%)	30 (76.9%)	24 (63.2%)
**Studies**		
Primary, n (%)	18 (46,2%)	8 (21,1%)
Middle, n (%)	19 (48,7%)	25 (65,8%)
Higher, n (%)	2 (5,13%)	5 (13,2%)
**Situación Laboral**		
Employed, n (%)	18 (46,2%)	13 (34,2%)
Unemployed, n (%)	21 (53,8%)	25 (65,8%)
**Smoking habits**		
Start smoking age, years	17.0 (15.0; 20.0)	16.0 (15.0; 18.0)
Cumulative consumption, pack-years	24.8 (16.6; 39.5)	21.2 (11.4; 35.5)
Current consumption, cigarettes/day	15.0 (10.0; 20.0)	12.0 (10.0; 20.0)
Current smoking, n (%)	39 (100%)	38 (100%)
